# Supplementary material for: Investigating the Endo-Lysosomal System in Major Neurocognitive Disorders Due to Alzheimer’s Disease, Frontotemporal Lobar Degeneration and Lewy Body Disease: Evidence for SORL1 as a Cross-Disease Gene
Source: Int J Mol Sci. 2021 Dec 20;22(24):13633. doi: 10.3390/ijms222413633 (PMC8704369; doi:10.3390/ijms222413633)
Supplement: Supplementary file 1 [file ijms-22-13633-s001.zip › TableS2_revised.pdf]

Table S2. List of 50 genes and functions reported by Gene Ontology.

Gene	Gene Ontology	
**ABCA2**	GO:0000166	enables nucleotide binding
	GO:0005319	enables lipid transporter activity
	GO:0005524	enables ATP binding
	GO:0016887	ATP hydrolysis activity
	GO:0042626	enables ATPase-coupled transmembrane transporter activity
	GO:0061135	enables endopeptidase regulator activity
	GO:0099038	enables ceramide floppase activity
	GO:0140359	enables ABC-type transporter activity
**AGRN**	GO:0002162	enables dystroglycan binding
	GO:0005200	enables structural constituent of cytoskeleton
	GO:0005201	enables extracellular matrix structural constituent
	GO:0005509	enables calcium ion binding
	GO:0005515	enables protein binding
	GO:0033691	enables sialic acid binding
	GO:0035374	enables chondroitin sulfate binding
	GO:0043236	enables laminin binding
	GO:0043395	enables heparan sulfate proteoglycan binding
**AP1B1**	GO:0005515	enables protein binding
	GO:0019901	enables protein kinase binding
	GO:0030276	enables clathrin binding
**AP1M1**	GO:0005515	enables protein binding
	GO:0035615	enables clathrin adaptor activity
**AP2A1**	GO:0005515	enables protein binding
	GO:0008022	enables protein C-terminus binding
	GO:0019901	enables protein kinase binding
	GO:0035615	contributes_to clathrin adaptor activity
	GO:0044877	enables protein-containing complex binding
	GO:0050750	enables low-density lipoprotein particle receptor binding
	GO:0140312	enables cargo adaptor activity
**AP2A2**	GO:0003674	enables molecular_function
	GO:0005515	enables protein binding
	GO:0008289	enables lipid binding
	GO:0019900	kinase binding
	GO:0019901	enables protein kinase binding
	GO:0019904	protein domain specific binding
	GO:0035615	contributes_to clathrin adaptor activity
	GO:0097718	enables disordered domain specific binding
	GO:0140312	enables cargo adaptor activity
**AP2B1**	GO:0005048	enables signal sequence binding
	GO:0005515	enables protein binding
	GO:0030276	enables clathrin binding
	GO:0035615	contributes_to clathrin adaptor activity
	GO:0044877	enables protein-containing complex binding
**AP3B2**	GO:0030276	clathrin binding
**AP3D1**	GO:0005515	enables protein binding
**ARRB1**	GO:0001664	enables G protein-coupled receptor binding
	GO:0003713	enables transcription coactivator activity
	GO:0004402	NOT enables histone acetyltransferase activity
	GO:0004857	enables enzyme inhibitor activity
	GO:0005096	enables GTPase activator activity
	GO:0005102	signaling receptor binding

	GO:0005159	enables insulin-like growth factor receptor binding
	GO:0005515	enables protein binding
	GO:0008134	enables transcription factor binding
	GO:0019899	enzyme binding
	GO:0030276	clathrin binding
	GO:0030331	enables estrogen receptor binding
	GO:0031625	enables ubiquitin protein ligase binding
	GO:0031691	enables alpha-1A adrenergic receptor binding
	GO:0031692	enables alpha-1B adrenergic receptor binding
	GO:0031701	enables angiotensin receptor binding
	GO:0031762	enables follicle-stimulating hormone receptor binding
	GO:0031896	enables V2 vasopressin receptor binding
	GO:0035612	enables AP-2 adaptor complex binding
	GO:0035615	enables clathrin adaptor activity
	GO:0044325	enables transmembrane transporter binding
	GO:0045309	enables protein phosphorylated amino acid binding
	GO:0051219	phosphoprotein binding
	GO:1990763	enables arrestin family protein binding
**ATP13A2**	GO:0000166	nucleotide binding
	GO:0005515	enables protein binding
	GO:0005524	enables ATP binding
	GO:0008270	enables zinc ion binding
	GO:0008289	lipid binding
	GO:0015417	enables ABC-type polyamine transporter activity
	GO:0016887	enables ATP hydrolysis activity
	GO:0019829	enables ATPase-coupled cation transmembrane transporter activity
	GO:0030145	enables manganese ion binding
	GO:0046872	metal ion binding
	GO:0070300	enables phosphatidic acid binding
	GO:0080025	enables phosphatidylinositol-3,5-bisphosphate binding
	GO:1903135	enables cupric ion binding
**ATP6V0A1**	GO:0005515	enables protein binding
	GO:0015078	enables proton transmembrane transporter activity
	GO:0046961	proton-transporting ATPase activity, rotational mechanism
	GO:0051117	enables ATPase binding
**ATP6V0D1**	GO:0005515	enables protein binding
	GO:0015078	proton transmembrane transporter activity
	GO:0044877	enables protein-containing complex binding
	GO:0046961	enables proton-transporting ATPase activity, rotational mechanism
**ATP6V1H**	GO:0005515	enables protein binding
	GO:0016887	contributes_to ATP hydrolysis activity
	GO:0030234	enables enzyme regulator activity
	GO:0046961	enables proton-transporting ATPase activity, rotational mechanism
**BCAN**	GO:0005515	enables protein binding
	GO:0005540	enables hyaluronic acid binding
	GO:0030246	enables carbohydrate binding
**CD63**	GO:0005515	enables protein binding
**CD81**	GO:0001618	enables virus receptor activity
	GO:0005178	enables integrin binding
	GO:0005515	enables protein binding
	GO:0008289	lipid binding
	GO:0015485	enables cholesterol binding
	GO:0023026	enables MHC class II protein complex binding
	GO:0042289	enables MHC class II protein binding
	GO:1990459	enables transferrin receptor binding
**CLTB**	GO:0005198	enables structural molecule activity
	GO:0005515	enables protein binding

	GO:0032050	enables clathrin heavy chain binding
	GO:0042277	enables peptide binding
CLTC	GO:0003723	enables RNA binding
	GO:0003725	enables double-stranded RNA binding
	GO:0005198	enables structural molecule activity
	GO:0005515	enables protein binding
	GO:0019901	enables protein kinase binding
	GO:0032051	enables clathrin light chain binding
	GO:0050750	enables low-density lipoprotein particle receptor binding
	GO:0097718	enables disordered domain specific binding
	GO:1990381	enables ubiquitin-specific protease binding
CORO1A	GO:0003723	enables RNA binding
	GO:0003779	enables actin binding
	GO:0003785	enables actin monomer binding
	GO:0005515	enables protein binding
	GO:0008022	enables protein C-terminus binding
	GO:0008092	enables cytoskeletal protein binding
	GO:0032036	enables myosin heavy chain binding
	GO:0042802	identical protein binding
	GO:0042803	enables protein homodimerization activity
	GO:0043548	enables phosphatidylinositol 3-kinase binding
	GO:0051015	enables actin filament binding
CTSA	GO:0004180	enables carboxypeptidase activity
	GO:0004185	enables serine-type carboxypeptidase activity
	GO:0008047	enables enzyme activator activity
	GO:0008233	peptidase activity
	GO:0016787	hydrolase activity
CTSD	GO:0004190	enables aspartic-type endopeptidase activity
	GO:0004197	enables cysteine-type endopeptidase activity
	GO:0005515	enables protein binding
	GO:0008233	enables peptidase activity
	GO:0016787	hydrolase activity
	GO:0070001	enables aspartic-type peptidase activity
DNAJC6	GO:0004721	phosphoprotein phosphatase activity
	GO:0004725	enables protein tyrosine phosphatase activity
	GO:0016787	hydrolase activity
	GO:0017124	enables SH3 domain binding
	GO:0030276	enables clathrin binding
DNM2	GO:0000166	nucleotide binding
	GO:0003924	enables GTPase activity
	GO:0005515	enables protein binding
	GO:0005525	enables GTP binding
	GO:0008017	enables microtubule binding
	GO:0016787	hydrolase activity
	GO:0017124	enables SH3 domain binding
	GO:0019899	enables enzyme binding
	GO:0019901	enables protein kinase binding
	GO:0031749	enables D2 dopamine receptor binding
	GO:0036312	enables phosphatidylinositol 3-kinase regulatory subunit binding
	GO:0044877	enables protein-containing complex binding
	GO:0050699	enables WW domain binding
	GO:0050998	enables nitric-oxide synthase binding
EHD3	GO:0000166	nucleotide binding
	GO:0003676	enables nucleic acid binding
	GO:0005509	enables calcium ion binding
	GO:0005515	enables protein binding
	GO:0005524	enables ATP binding
	GO:0005525	enables GTP binding

	GO:0046872	metal ion binding
GGA1	GO:0003674	enables molecular_function
	GO:0005515	enables protein binding
	GO:0031267	enables small GTPase binding
	GO:0035091	enables phosphatidylinositol binding
	GO:0043130	enables ubiquitin binding
GGA2	GO:0005515	enables protein binding
	GO:0031267	enables small GTPase binding
	GO:0035091	enables phosphatidylinositol binding
	GO:0043130	enables ubiquitin binding
GGA3	GO:0005515	enables protein binding
	GO:0031267	enables small GTPase binding
	GO:0035091	enables phosphatidylinositol binding
	GO:0043130	enables ubiquitin binding
	GO:0044877	enables protein-containing complex binding
GNPTG	GO:0003976	enables UDP-N-acetylglucosamine-lysosomal-enzyme N-acetylglucosaminephosphotransferase activity
	GO:0042803	enables protein homodimerization activity
GPC1	GO:0005507	enables copper ion binding
	GO:0017134	enables fibroblast growth factor binding
	GO:0043236	enables laminin binding
GPRASP1	GO:0005515	enables protein binding
HGS	GO:0005515	enables protein binding
	GO:0016301	kinase activity
	GO:0019904	enables protein domain specific binding
	GO:0035091	enables phosphatidylinositol binding
	GO:0043130	enables ubiquitin binding
	GO:0044389	enables ubiquitin-like protein ligase binding
	GO:0046872	enables metal ion binding
HSP90AA1	GO:0000166	nucleotide binding
	GO:0003723	enables RNA binding
	GO:0005515	enables protein binding
	GO:0005524	enables ATP binding
	GO:0016787	hydrolase activity
	GO:0016887	enables ATP hydrolysis activity
	GO:0023026	enables MHC class II protein complex binding
	GO:0030235	enables nitric-oxide synthase regulator activity
	GO:0030911	enables TPR domain binding
	GO:0031625	enables ubiquitin protein ligase binding
	GO:0042802	enables identical protein binding
	GO:0042803	enables protein homodimerization activity
	GO:0042826	enables histone deacetylase binding
	GO:0048156	enables tau protein binding
	GO:0051020	enables GTPase binding
	GO:0051082	enables unfolded protein binding
	GO:0070182	enables DNA polymerase binding
	GO:0097110	enables scaffold protein binding
	GO:0097718	enables disordered domain specific binding
	GO:1990782	enables protein tyrosine kinase binding
HSPA8	GO:0000166	nucleotide binding
	GO:0001664	enables G protein-coupled receptor binding
	GO:0001786	enables phosphatidylserine binding
	GO:0003723	enables RNA binding
	GO:0005515	enables protein binding
	GO:0005524	enables ATP binding
	GO:0016787	hydrolase activity
	GO:0016887	enables ATP hydrolysis activity

	GO:0019899	enables enzyme binding
	GO:0023026	enables MHC class II protein complex binding
	GO:0030674	enables protein-macromolecule adaptor activity
	GO:0031072	enables heat shock protein binding
	GO:0031625	enables ubiquitin protein ligase binding
	GO:0044183	enables protein folding chaperone
	GO:0045296	enables cadherin binding
	GO:0051082	enables unfolded protein binding
	GO:0051087	enables chaperone binding
	GO:0051787	enables misfolded protein binding
	GO:0055131	enables C3HC4-type RING finger domain binding
	GO:0140597	enables protein carrier chaperone
	GO:1990833	enables clathrin-uncoating ATPase activity
**LAMP1**	GO:0001618	enables virus receptor activity
	GO:0005515	enables protein binding
	GO:0019899	enables enzyme binding
	GO:0019904	enables protein domain specific binding
**LAPTM4B**	GO:0005515	enables protein binding
	GO:0019900	enables kinase binding
	GO:0031625	enables ubiquitin protein ligase binding
	GO:0097001	enables ceramide binding
	GO:1902936	enables phosphatidylinositol bisphosphate binding
**MGRN1**	GO:0004842	enables ubiquitin-protein transferase activity
	GO:0005515	enables protein binding
	GO:0016740	transferase activity
	GO:0046872	enables metal ion binding
	GO:0061630	enables ubiquitin protein ligase activity
**NCAN**	GO:0005509	enables calcium ion binding
	GO:0005540	enables hyaluronic acid binding
	GO:0030246	enables carbohydrate binding
**NEU1**	GO:0004308	enables exo-alpha-sialidase activity
	GO:0005515	enables protein binding
	GO:0016787	hydrolase activity
	GO:0016798	hydrolase activity, acting on glycosyl bonds
	GO:0016997	enables alpha-sialidase activity
	GO:0052794	enables exo-alpha-(2->3)-sialidase activity
	GO:0052795	enables exo-alpha-(2->6)-sialidase activity
	GO:0052796	enables exo-alpha-(2->8)-sialidase activity
**PPT1**	GO:0005515	enables protein binding
	GO:0008474	enables palmitoyl-(protein) hydrolase activity
	GO:0016290	enables palmitoyl-CoA hydrolase activity
	GO:0016787	hydrolase activity
	GO:0016790	enables thiolester hydrolase activity
	GO:0035727	enables lysophosphatidic acid binding
	GO:0098599	palmitoyl hydrolase activity
	GO:0120146	enables sulfatide binding
**PSAP**	GO:0002020	enables protease binding
	GO:0005515	enables protein binding
	GO:0005543	enables phospholipid binding
	GO:0008047	enables enzyme activator activity
	GO:0042802	identical protein binding
	GO:0042803	enables protein homodimerization activity
	GO:0097110	enables scaffold protein binding
	GO:1905573	enables ganglioside GM1 binding
	GO:1905574	enables ganglioside GM2 binding
	GO:1905575	enables ganglioside GM3 binding
	GO:1905576	enables ganglioside GT1b binding
	GO:1905577	enables ganglioside GP1c binding

**RAB12**	GO:0003924	enables GTPase activity
	GO:0005515	enables protein binding
	GO:0005525	enables GTP binding
	GO:0019003	enables GDP binding
**RAB7A**	GO:0000166	nucleotide binding
	GO:0003924	enables GTPase activity
	GO:0003925	enables G protein activity
	GO:0005515	enables protein binding
	GO:0005525	enables GTP binding
	GO:0016787	hydrolase activity
	GO:0019003	enables GDP binding
	GO:1905394	enables retromer complex binding
**SH3GL2**	GO:0005515	enables protein binding
	GO:0008022	enables protein C-terminus binding
	GO:0008289	enables lipid binding
	GO:0019901	enables protein kinase binding
	GO:0042802	enables identical protein binding
**SORL1**	GO:0001540	enables amyloid-beta binding
	GO:0004888	enables transmembrane signaling receptor activity
	GO:0005041	enables low-density lipoprotein particle receptor activity
	GO:0005515	enables protein binding
	GO:0030169	enables low-density lipoprotein particle binding
	GO:0031267	enables small GTPase binding
	GO:0042923	enables neuropeptide binding
**TOM1**	GO:0005515	enables protein binding
	GO:0030276	enables clathrin binding
	GO:0035091	enables phosphatidylinositol binding
	GO:0043130	enables ubiquitin binding
**USP5**	GO:0004197	enables cysteine-type endopeptidase activity
	GO:0004843	enables thiol-dependent deubiquitinase
	GO:0005515	enables protein binding
	GO:0008233	peptidase activity
	GO:0008234	cysteine-type peptidase activity
	GO:0008270	enables zinc ion binding
	GO:0016787	hydrolase activity
	GO:0043130	enables ubiquitin binding
	GO:0046872	metal ion binding
**VPS16**	GO:0003779	enables actin binding
	GO:0005515	enables protein binding
	GO:0051015	enables actin filament binding
**VPS39**	GO:0005515	enables protein binding
**VPS52**	GO:0005515	enables protein binding
	GO:0019905	enables syntaxin binding
