# Supplementary material for: Investigating the Endo-Lysosomal System in Major Neurocognitive Disorders Due to Alzheimer’s Disease, Frontotemporal Lobar Degeneration and Lewy Body Disease: Evidence for SORL1 as a Cross-Disease Gene
Source: Int J Mol Sci. 2021 Dec 20;22(24):13633. doi: 10.3390/ijms222413633 (PMC8704369; doi:10.3390/ijms222413633)
Supplement: Supplementary file 1 [file ijms-22-13633-s001.zip › TableS3_revised.pdf]

Table S3. List of 50 genes selected with bioinformatics tools with reports of association to neurodegenerative diseases and others neurological disorders from literature

Gene	Neurodegenerative Diseases (AD, DLB, FTLD, PD)	Others Neurological disorders
**ABCA2**	AD (Genetic and molecular studies in humans and cellular models)<br>doi:10.2174/1567205012091510191O5834<br>doi:10.1016/j.nbd.2004.09.011<br>doi:10.1074/jbc.M111.288258	-
**AGRN**	AD (Molecular studies in animal models)<br>doi:10.1097/NEN.0b013e31823b0b12	Congenital myasthenic syndrome (Genetic studies in humans)<br>doi:10.1007/s00439-011-1132-4
**AP1B1**	-	-
**AP1M1**	-	-
**AP2A1**	-	Sporadic Creutzfeldt-Jakob disease (Molecular studies in humans)<br>doi:10.1007/s12035-016-0029-6
**AP2A2**	AD (Genetic and molecular studies in humans)<br>doi:10.1093/jnen/nlz116<br>doi:10.1038/ng.2802	-
**AP2B1**	PD (Molecular studies in humans)<br>doi:10.1186/s13195-019-0533-9	
**AP3B2**	-	Epileptic Encephalopathy (Genetic studies in humans)<br>doi:10.1016/j.ajhg.2016.10.009
**AP3D1**	-	Hermansky-Pudlock syndrome (Genetic studies in humans)<br>doi:10.1016/j.ejmg.2018.11.017
**ARRB1**	AD (Molecular studies in humans)<br>doi:10.1038/s41598-017-01798-x	-
**ATP13A2**	PD (Genetic studies in humans)<br>doi:10.1001/archneurol.2010.281<br>doi:10.1371/journal.pgen.1007045	-
**ATP6V0A1**	AD (Molecular studies in humans and animal models)<br>doi:10.1038/s41467-021-22389-5<br>doi:10.1016/j.cell.2010.05.008<br>PD (Genetic studies in humans)<br>doi: 10.1016/S1474-4422(19)30320-5	-
**ATP6V0D1**	AD (Molecular studies in humans)<br>doi:10.1093/brain/awaa223	-
**ATP6V1H**	AD (Genetic and molecular studies in humans)<br>doi:10.1186/s12881-018-0603-z<br>doi:10.1155/2021/9918498	-
**BCAN**	AD (Molecular studies in animal models)<br>doi:10.1111/j.1471-4159.2010.06647.x	-
**CD63**	-	-
**CD81**	-	-
**CLTB**	-	-
**CLTC**	AD (Molecular studies in humans and animal models)<br>doi:10.1007/s12539-019-00353-8<br>PD (Genetic studies in humans)<br>doi:10.1016/j.parkreldis.2018.10.012	Developmental Delay (Genetic studies in humans)<br>doi:10.1002/ajmg.a.37506

**CORO1A**	-	-
**CTSA**	-	Galactosialidosis<br>(Genetic studies in humans)<br>doi:10.1093/hmg/5.12.1977
**CTSD**	-	-
**DNAJC6**	PD (Genetic studies in humans)<br>doi:10.1186/s13024-021-00455-2<br>doi:10.1016/j.parkreldis.2012.11.006<br>doi:10.1002/ana.24553	-
**DNM2**	AD (Genetic and molecular studies in humans and animal models)<br>doi:10.1007/s10038-008-0251-9<br>doi:10.1016/j.bbrc.2008.12.147<br>doi:10.5607/en.2019.28.3.390	Charcot-Marie-Tooth disease (Genetic studies in humans)<br>doi:10.1007/s10072-020-04972-8
**EHD3**	AD (Molecular studies in cellular models)<br>doi:10.1016/j.celrep.2013.12.006	-
**GGA1**	AD (Molecular studies in humans and cellular models)<br>doi:10.1091/mbc.E12-01-0014<br>doi:10.1371/journal.pone.0129047	-
**GGA2**	AD (Molecular studies in humans and cellular models)<br>doi:10.1371/journal.pone.0129047	-
**GGA3**	AD (Molecular studies in humans and cellular models)<br>doi:10.1016/j.neuron.2007.05.012<br>doi:10.1371/journal.pone.0129047	-
**GNPTG**	-	Mucolipidosis type III<br>(Genetic and molecular studies in humans)<br>doi:10.1002/humu.20959
**GPC1**	AD (Molecular studies in humans)<br>doi:10.1096/fj.03-1040fje	-
**GPRASP1**	-	-
**HGS**	-	-
**HSP90AA1**	-	-
**HSPA8**	AD (Molecular studies in humans)<br>doi:10.3233/JAD-130428<br>PD (Genetic studies in humans)<br>doi:10.1186/s13024-021-00455-2	-
**LAMP1**	AD (Molecular studies in humans and animal models)<br>doi:10.1186/s13024-021-00464-1	-
**LAPTM4B**	DLB (Genetic studies in humans)<br>doi:10.1016/j.neurobiolaging.2018.10.019	-
**MGRN1**	PD (Molecular studies in animal models)<br>doi:10.1126/science.1079694	-
**NCAN**	PD (Molecular studies in animal models)<br>doi:10.1016/j.neulet.2020.135074	-
**NEU1**	AD (Molecular studies in humans and animal models)<br>doi:10.1038/ncomms3734<br>doi:10.3233/JAD-201039	Sialidosis (Genetic and molecular studies in humans and cellular models)<br>doi:10.1371/journal.pone.0104229<br>doi:10.1093/hmg/9.18.2715
**PPT1**	AD (Molecular studies in animal models)<br>doi:10.1016/j.nbd.2019.104603<br>FTD (Molecular studies in animal models)<br>doi:10.1186/s40478-020-01037-x	Neuronal Ceroid Lipofuscinosis (Genetic and molecular studies in humans)<br>doi:10.1002/ana.1103;<br>doi: 10.1006/mgme.1999.2803.

**PSAP**	AD (Molecular studies in humans and animal models)<br>doi:10.1016/j.cca.2019.03.243<br>doi:10.1186/s13024-021-00464-1<br>PD (Genetic studies in humans)<br>doi:10.1093/brain/awaa064	-
**RAB12**	-	-
**RAB7A**	AD (Molecular studies in humans)<br>doi:10.1038/s41380-019-0602-2	Charcot-Marie-Tooth Type 2B Neuropathy (Genetic studies in humans)<br>doi:10.1086/367847
**SH3GL2**	PD (Genetic studies in humans)<br>doi:10.1212/NXG.0000000000000557<br>AD (Molecular studies in humans)<br>doi:10.1080/00207454.2020.1860966	-
**SORL1**	AD (Genetic and molecular studies in humans)<br>doi:10.1001/archneurol.2011.788<br>doi:10.1186/s13195-016-0222-x<br>doi:10.1038/ng1943<br>doi:10.1002/ana.24305<br>FTLD (Genetic studies in humans)<br>doi:10.3390/ijms20163903<br>PD (Genetic studies in humans)<br>doi:10.1002/acn3.51433	-
**TOM1**	AD (Molecular studies in humans)<br>doi:10.1016/j.jns.2016.03.035	Immunodeficiency and autoimmune disease (Genetic studies in humans)<br>doi:10.1038/s41525-019-0088-5
**USP5**	-	-
**VPS16**	-	Dystonia (Genetic studies in humans)<br>doi:10.1002/ana.25879
**VPS39**	-	Schizophrenia (Genetic studies in humans)<br>doi:10.1038/ng.2446
**VPS52**	PD (Molecular studies in animal and cellular models)<br>doi:10.1016/j.celrep.2020.107614	-
